# Phenotypic characterization of *Gardnerella vaginalis* subgroups suggests differences in their virulence potential

**DOI:** 10.1371/journal.pone.0200625

**Published:** 2018-07-12

**Authors:** Migle Janulaitiene, Vilmantas Gegzna, Lina Baranauskiene, Aistė Bulavaitė, Martynas Simanavicius, Milda Pleckaityte

**Affiliations:** 1 Institute of Biotechnology, Vilnius University, Vilnius, Lithuania; 2 National Public Health Surveillance Laboratory, Vilnius, Lithuania; 3 Institute of Photonics and Nanotechnology, Vilnius University, Vilnius, Lithuania; 4 Institute of Biosciences, Vilnius University, Vilnius, Lithuania; Massachusetts General Hospital, UNITED STATES

## Abstract

The well-known genotypic and phenotypic diversity of *G*. *vaginalis* resulted in its classification into at least four subgroups (clades) with diverse genomic properties. To evaluate the virulence potential of *G*. *vaginalis* subgroups, we analyzed the virulence-related phenotypic characteristics of 14 isolates of clade 1, 12 isolates of clade 2, 8 isolates of clade 4 assessing their *in vitro* ability to grow as a biofilm, produce the toxin vaginolysin, and express sialidase activity. Significant differences in VLY production were found (*p* = 0.023), but further analysis of clade pairs did not confirm this finding. The amount of biofim did not differ significantly among the clades. Analysis of sialidase activity indicated statistically significant differences among the clades (*p* < 0.001). Production of active recombinant *G*. *vaginalis* sialidase demonstrated the link between the *sld* gene and enzymatic activity, which may be differentially regulated at the transcriptional level. Statistical classification analysis (random forests algorithm) showed that *G*. *vaginalis* clades could be best defined by the profiles of two phenotypic characteristics: sialidase activity and vaginolysin production. The results of principal component analysis and hierarchical clustering suggested that all isolates can be subgrouped into three clusters, the structures of which are determined based on phenotypic characteristics of the isolates. Clade 4 was the most homogenous group, as all isolates were found in the same cluster, which is characterized by low production of all studied virulence factors. Clade 2 isolates were mainly distributed between two clusters, whereas clade 1 isolates were found in all three clusters that were characterized by a distinct profile of phenotypic characteristics. Our findings suggest that *G*. *vaginalis* subgroups with different virulence potential might play distinct roles in vaginal microbiota.

## Introduction

The bacterium *Gardnerella vaginalis* is a member of vaginal microbial communities of healthy women and those with bacterial vaginosis [1, 2]. The characteristic feature of BV is a loss of vaginal lactobacilli and an overgrowth of diverse anaerobes [2]. To reflect the recognized phenotypic and genotypic heterogeneity of *G*. *vaginalis*, biotyping and genotyping methods were developed [3, 4]. However, these early typing assays had a limited success in revealing *G*. *vaginalis* diversity [5]. Recent study has approach this problem by sequencing of the gene encoding chaperonin 60 (*cpn60*) [6]. Four subgroups (A–D) in a group of 112 *G*. *vaginalis* isolates have been distinguished using this method [7]. Analysis of whole-genome sequences differentiated *G*. *vaginalis* strains into four clades (1–4) [8]. It was shown that the four cpn60-based subgroups (A, B, C, and D) correspond to clades 4, 2, 1, and 3, respectively [7]. It appears that the four-group division might be expanded, as subtyping of *G*. *vaginalis* clinical isolates indicated the presence of the previously unknown subgroups [7, 9].

*Gardnerella vaginalis* exhibits exceptional virulence potential compared with other BV-associated bacteria and establishes a specific interaction with vaginal microbial species [10–12]. It was demonstrated that *G*. *vaginalis* secretes the cholesterol-dependent toxin vaginolysin, which pore-forming activity is associated with cytotoxicity [12, 13]. Sialidase produced by *G*. *vaginalis* participates in the degradation of mucosal sialoglycans, that is believed to be important in BV [11, 14, 15]. *G*. *vaginalis* is able to adhere to vaginal epithelial cells and produce a biofilm [10]. Structured polymicrobial biofilm consisting of numerically predominant *G*. *vaginalis* and other incorporated bacterial species was detected in BV patients [16, 17]. Previous studied showed that the virulence-related phenotypic characteristics were differentially expressed across *G*. *vaginalis* isolates [18–20]. It has to be determined whether the relationship between these characteristics and the sequence-based genotyping subgroups exists.

Initial studies demonstrated that isolates of clade 4 do not possess the gene encoding sialidase [7, 9]. Sialidase activity was detected in all isolates of subgroup B (corresponding to clade 2) and some subgroup C isolates (clade 1) but not isolates of subgroups A (clade 4) or D (clade 3) [7]. Moreover, subgroup B (clade 2) exhibited higher sialidase activity than other subgroups [7].

In this study, we evaluated the genotypic and phenotypic characteristics associated with the virulence of three subgroups of *G*. *vaginalis*. To assess distribution of virulence factors across the subgroups, clinical isolates of clades 1, 2, and 4 were characterized based on their ability to produce the toxin vaginolysin (VLY), grow as a biofilm, and express sialidase activity. We also produced *G*. *vaginalis–*derived recombinant sialidase and characterized its enzymatic activity in order to determine the relationship between the presence of the sialidase-encoding gene and sialidase activity.

## Materials and methods

### Ethics statement

In the present study we characterized the clinical isolates obtained from the previous study, results of which were published in Janulaitiene et al., BMC Infect Dis, 2017; doi: 10.1186/s12879-017-2501-y. The previous study (Janulaitiene et al., 2017) was approved by the Lithuanian Bioethics Committee (approval no. 158200-1-3697-223, 12/11/2013; amendment no. 2, 8/12/2015). Written informed consent was obtained from all study participants prior to enrollment.

In the present study there is no involvement of human participants, specimens or tissue samples.

### *Gardnerella vaginalis* clinical isolates

Thirty-five *Gardnerella vaginalis* isolates were obtained from previously characterized cultured vaginal samples of women from Lithuania [9]. *Gardnerella vaginalis* strain 14018 was obtained from the American Type Culture Collection (ATCC).

### Cultivation of *G*. *vaginalis* isolates

Frozen stocks in tryptic soy broth (TSB) supplemented with 20% horse serum (Oxoid) and 15% glycerol were recovered on chocolate agar (Oxoid) for 48 h at 37°C and 5% CO_2_. Cultures were inoculated in 7 mL of liquid medium (TSB supplemented with 0.15% soluble starch and 2% horse serum) and grown for 24 h at 37°C and 5% CO_2_. The 24-h bacterial cultures were then adjusted to 2×10^6^ CFU/mL in 7 mL of fresh medium and grown for 24 h at 37°C and 5% CO_2_. Optical density at 600 nm (OD_600_) was measured before culture harvesting. The growth of each isolate in liquid medium was assayed in duplicate in three independent experiments. Cells were collected by centrifugation; supernatants were filtered through 0.22-μm membrane filters (Roth), aliquoted into portions, and stored frozen. Those supernatants were used for sialidase activity and VLY quantification assays.

### DNA isolation, genotyping, and gene-specific PCR

DNA was isolated from harvested cells using a GeneJET genomic DNA purification kit (Thermo Fisher Scientific, Vilnius, Lithuania) according to the manufacturer’s instructions. *Gardnerella vaginalis* isolates were subtyped using clade-specific single-plex PCR, as described previously [9, 21]. Amplified ribosomal DNA restriction analysis (ARDRA) genotyping was performed by amplification of the 16S rRNA gene with subsequent digestion of the PCR product with TaqI restriction endonuclease as described previously [4]. The gene encoding sialidase A (*sld*) was detected using two sets of primers [9]. The presence of the VLY-encoding gene (*vly*) was detected using the primers VLY-585F and VLY-1334R [9]. Where indicated, the flanking regions of the *vly* gene were amplified, as described elsewhere [22]. The sequences of all primers used in this study are provided in S1 Table.

### Quantification of VLY

VLY was quantified using a monoclonal antibody (MAb)-based sandwich ELISA, as described previously [19]. Briefly, microtiter plates (Nunc MaxiSorp, Roskilde, Denmark) were coated with MAb 12E1 (5 μg/mL) [23] and then blocked by incubation with Roti-Block solution (Carl Roth, Karlsruhe, Germany). For preparation of a calibration curve, purified recombinant VLY [23] was added to the wells at concentrations ranging from 1 ng/mL to 1 μg/mL. Supernatants of *G*. *vaginalis* cultures were thawed and serially diluted in phosphate-buffered saline (PBS) containing 0.1% Tween 20 and applied to the wells for VLY quantification. After incubation and washing steps, the plates were incubated with horseradish peroxidase–conjugated (Bio-Rad, Hercules, CA) MAb 9B4 [23] (prepared in house) at a dilution of 1:4000. The enzymatic reaction was developed using ready-to-use tetramethylbenzidine substrate (Sigma-Aldrich) and stopped by adding 3.6% H_2_SO_4_. OD was measured using a Multiskan GO microplate spectrophotometer (Thermo Fisher Scientific, Waltham, MA, USA) and calculated as the difference between OD values at 450 nm and 620 nm. The concentration of VLY in the test samples was calculated from the linear part of the calibration curve. VLY quantification assays for each supernatant were performed in triplicate. VLY concentration was normalized to the OD of the cultures, and the results were averaged.

### Biofilm assay

*Gardnerella vaginalis* clinical isolates and strain ATCC 14018 were grown in biofilm medium BHIs (brain-heart infusion broth supplemented with 2% horse serum, 0.15% soluble starch, and 1% glucose) for 24 h at 37°C and 5% CO_2_. Overnight bacterial cultures were adjusted to 3×10^7^ CFU/mL in fresh biofilm medium. A volume of 150 μL was added to sterile 96-well polystyrene tissue culture plates (Orange Scientific) and incubated for 24 and 48 h at 37°C and 5% CO_2_. As a negative control, four wells of each microplate were filled with medium without bacteria. After incubation, spent medium was removed, and the wells were washed three times with PBS to remove non-adherent bacteria. The plates were then air dried. Adherent cells were stained with 150 μL of safranine (Sigma) for 15 min. Wells were washed twice with 150 μL of PBS to remove excess safranin, and the plates were air dried in the dark. Safranin was solubilized by adding 150 μL of 33% acetic acid per well. From each well, 75 μL was transferred to a new plate, and the OD_492_ was measured on a microplate reader (Anthos 2020). At least four independent experiments on different days with eight technical replicates were performed for each *G*. *vaginalis* isolate and strain ATCC 14018. For each sample, the average OD_492_ form the negative controls (containing no cells) were subtracted from the OD_492_ of the test wells before statistical evaluation. Based on the OD_492_ data, the isolates were classified into categories as described previously [24]. The cut-off OD (ODc) was defined as three standard deviations (SDs) above the mean OD of the negative control. Strains were classified into the four categories according to the following criteria: no biofilm formers when OD≤ODc, weak biofilm formers when ODc<OD≤2×ODc, moderate biofilm formers when 2×ODc<OD≤4×ODc, strong biofilm formers when 4×ODc<OD.

### Quantification of sialidase activity

The supernatants of *G*. *vaginalis* cultures were thawed and diluted 20-fold with 50 mM sodium acetate (pH 5.5) containing 200 mM 4-methylumbelliferyl-α-D-*N*-acetylneuraminic acid sodium salt hydrate (MUN) (Sigma-Aldrich, St. Louis, MO). Reaction mixture (100 μL volume/well) was applied to the wells of black polystyrene microplates (Nunc, Thermo Scientific). The plates were sealed with an optically clear seal, and MUN hydrolysis was monitored by measuring the fluorescence at an excitation wavelength of 365 nm (bandwidth 9 nm) and emission wavelength of 440 nm (bandwidth 20 nm) with gain = 85 every 2 min using a Synergy H4 hybrid multi-mode microplate reader (Biotek, Winooski, VT, USA) at 30°C with continuous shaking at 17 Hz. The fluorescence of each supernatant was analyzed in duplicate. Relative sialidase activity was determined as the slope of the change in fluorescence over the initial approximately linear increase in fluorescence. Sialidase activity was normalized to the OD of bacterial cultures.

### Cloning and expression of the sialidase gene from *G*. *vaginalis*

Purified genomic DNA from *G*. *vaginalis* isolates 47.3, 58.4, 60.1, 79.2, 86.1, and 114.2 was used to amplify the full-length *sld* gene with primers Sia5-F and Sia3-R with Phusion Flash high-fidelity master mix (Thermo Fisher Scientific). Primers SiaK-F and Sia3-R were designed to amplify the sialidase catalytic domain using genomic DNA extracted from isolates 86.1 and 114.2. The sequences of all primers dedicated to amplification of the *sld* gene are provided in S1 Table. The resulting PCR products were verified by sequencing. The sialidase-encoding DNA fragments were digested with BcuI and SalI restriction endonucleases and cloned into NheI/SalI–digested pET28a(+) vector (Novagen, Merck). The resulting constructs included the *sld* gene fused with an N-terminal hexahistidine tag. The pET28 plasmids bearing the *sld* genes were transformed into *E*. *coli* Tuner (DE3) (Novagen, Merck). Synthesis of the recombinant proteins was induced with 0.1 mM isopropyl β-D-1-thiogalactopyranoside (Thermo Fisher Scientific). After 4 h of induction at 25°C, the cells were pelleted, disrupted by sonication, and centrifuged at 3200 *g* for 15 min. The cell-free fraction was filtered through a 0.22-μm membrane, and the cell pellet was analyzed by 10% sodium dodecyl sulfate–polyacrylamide gel electrophoresis (SDS-PAGE) under reducing conditions. Protein concentrations in the filtered fractions were measured using the Bradford assay (Roth, Karlsruhe, Germany). The prepared supernatants were aliquoted into portions and stored frozen.

Activity of recombinant sialidase in the filtered soluble fraction of *E*. *coli* lysate was measured using the fluorogenic substrate MUN, as described above. Samples diluted 20- and 50-fold were prepared, and the fluorescence of each sample (at the respective dilution) was analyzed in duplicate. Relative sialidase activity was normalized to the total protein concentration of the sample. Non-transformed *E*. *coli* Tuner (DE3) cells were used as a negative control. At least two clones producing each recombinant sialidase were subjected to the enzymatic activity assay, and the results were averaged.

### Statistical analysis

The data were analyzed using the R statistical computer program, version 3.4.3 [25]. The dataset subjected for analysis consisted of log-transformed numeric phenotypic characteristics (VLY production, biofilm amount expressed as OD, and sialidase activity) and the clades. Logarithmic transformations were applied as follows: (i) x’ = log(x), where x is the amount of biofilm; (ii) y’ = log(y + 1), where y is either sialidase activity or VLY amount (a value of ‘1’ had to be added where values of ‘0’ were present).

Differences in the expression of phenotypic characteristics across *G*. *vaginalis* clades were evaluated either by (a) Welch F test [26] followed by Games-Howell [27] *post hoc* procedure for pairwise comparisons [27] for approximately normal data with significantly different variances (Brown-Forsythe test [28]) or (b) Kruskal-Wallis test [26] followed by Conover-Iman *post hoc* procedure for pairwise comparisons [29], with Holm-method–corrected *p* values [26] for data with strongly violated normality assumptions. The results of statistical comparisons are reported as the test statistic (*H*, *F*, *t*) value with its parameters in parentheses and associated *p* value. A significance level of α = 0.05 (*p <* 0.05) was considered statistically significant. The results of *post hoc* pairwise comparisons were expressed as a form of compact letter display (CLD) [30]. CLD indicates that there are no statistically significant differences (*p* < 0.05) between a pair of compared groups if the groups share the same CLD letter. The assumption of normality was evaluated using the Shapiro-Wilk test of normality [26], with the significance level set to 0.01.

Associations among the numeric phenotypic characteristics were investigated using Spearman’s rank correlation analysis [26]. Statistical significance of the correlation was tested (*p* values were corrected using Holm’s procedure), and the 95% confidence interval (CI) of this coefficient was approximated using the bias-corrected and accelerated method [31] based on 10^4^ bootstrap resamples.

Classification properties of every numeric characteristic (predictor) and each combination of the predictors to identify the three clades of *G*. *vaginalis* were analyzed using a random forests algorithm (implemented in the R package “ranger” [32]) with 10^4^ trees. Hyper-parameter tuning and best model selection were based on a stratified 100-times repeated 3-fold cross-validation performed through the interface of the R package “caret” [33] using Cohen’s kappa coefficient [34] as a performance measure. The kappa values can be interpreted as follows: 1, perfect classification; 0, chance-level classification. The performance of each combination of predictors was evaluated indicating mean kappa and its 95% CI based on 300 cross-validation resamples. Pairwise comparisons of models were performed using the paired-samples Student’s *t*-test [26] with Bonferroni procedure [26] for *p* value correction.

Other possible relationships between the clades and the phenotypic characteristics were explored using principal component analysis (PCA) [26] and hierarchical clustering (HC) applied to the mean-centered and SD-scaled (z-transformed) data. The HC analysis was based on the Euclidean distance and Ward’s linkage method [35]. The results were visualized by means of dendograms and a PCA biplot of the first two principal components (PCs). The presence of three clades suggested that the data be divided into three clusters. The clusters were determined by selecting the three largest branches in the dendogram. The Calinski-Harabasz (CH) index [36] was used as an additional criterion to evaluate the quality of clustering (i.e., clusters with a higher CH index are preferred).

## Results

### Collection of *G*. *vaginalis* isolates

*G*. *vaginalis* clinical strains isolated from 23 characterized vaginal samples from Lithuanian women [9] were subtyped on the basis of clade-specific genes, as described previously [9, 21]. Fourteen isolates were defined as belonging to clade 1, 12 isolates belonged to clade 2, and 8 isolates belonged to clade 4. We found no clade 3 *G*. *vaginalis* strains among those collected. Isolate 86.1 was negative in all clade-specific PCR assays and designated of an unknown clade [9]. Two isolates (99.1 and 105.1) originated from vaginal samples with normal vaginal flora (NS = 3), whereas the other 33 isolates were from samples with a NS ≥4 (Table 1).

**Table 1:** Genotypic and phenotypic characteristics of *G*. *vaginalis* isolates.

Vaginal sample	Nugent score	Clade in vaginal sample	Isolate	Clade of isolate	ARDRA genotype	Vaginolysin (VLY)		Biofilm former^b^	Sialidase	
						Gene	VLY production^a^		Gene	Sialidase activity^c^
046S1	7	1+2+4	46.6	1	2	+	132.7± 20.9	weak	+	0
047S1	10	1+4	47.3	1	2	+	125.6 ± 7.9	weak	+	9450 ± 305
056S1	6	1+4	56.1	1	2	+	270.3 ± 6.6	strong	+	0
057S1	10	1	57.1	1	2	+	381.2 ± 36.6	weak	+	30 ± 6
058S1	9	1+2+4	58.1	4	1	+	3.71 ± 0.33	non	−	0
			58.4	1	2	+	1900.8 ± 89.7	moderate	+	1628 ± 70
			58.7	2	1	−	0	non	+	525 ± 28
058S2	5	4	58.2.1	4	1	+	2.0 ± 0.2	non	−	0
			58.2.3	1	2	+	2062.5 ± 147.9	moderate	+	0
060S1	10	2+4	60.1	2	1	+	101.5 ± 5.1	strong	+	34 ± 3
063S1	8	1+2+4	63.1	4	1	+	0	non	−	0
			63.2	2	1	+	1809.8 ± 161.3	strong	+	362 ± 158
065S1	9	1+2+4	65.2	2	1	+	1151.6 ± 12.7	non	+	867 ± 54
076S1	6	1+2+4	76.2	1	2	+	61.9 ± 2.9	strong	+	13 ± 2
078S1	5	2+4	78.1	2	1	+	141.0 ± 32.6	weak	+	250 ± 68
079S1	4	1	79.2	1	2	+	20.7 ± 4.5	moderate	+	0
082S1	9	1+2+4	82.1	4	1	+	7.6 ± 0.5	non	−	0
			82.2	2	1	+	56.6 ± 4.7	strong	+	43 ± 18
083S1	6	1+2	83.1	1	2	+	126.8 ± 25.4	weak	+	5 ± 1
084S1	9	1+2+4	84.1	1	2	+	105.0 ± 24.9	weak	+	4 ± 1
			84.3	2	1	−	0	non	+	23 ± 7
			84.4	2	1	−	0	weak	+	455 ± 126
			84.5	1	2	+	67.4 ± 9.8	moderate	+	24 ±4
			84.6	2	1	−	0	weak	+	317 ± 40
086S1	6	1+2+3+4	86.1	ND	2	−	0	moderate	+	0
			86.3	2	2	+	743.8 ± 49.2	strong	+	727 ± 41
			86.5	2	1	−	0	weak	+	550 ± 24
088S1	10	1+2+3+4	88.2	4	1	+	0	weak	−	0
099S1	3	1+2+3+4	99.1	4	1	+	44.1 ± 12.3	weak	−	0
103S1	5	2+4	103.1	2	1	+	14.1 ± 1.6	weak	+	55 ± 15
105S1	3	1+4	105.1	1	2	+	68.0 ± 5.3	weak	+	0
106S1	9	1+2+3+4	106.3	4	1	+	797.8 ± 90.9	moderate	−	0
			106.5	1	2	+	213.6 ± 21.5	strong	+	121 ± 7
107S1	10	1+2+4	107.1	4	1	+	298.8 ± 28.5	weak	−	0
114S1	10	1+2+4	114.2	1	2	+	192.2 ± 25.2	strong	+	36 ± 2

^a^Values are VLY concentration (ng/mL) normalized to OD_600_ of the cultures expressed as the mean of three biological replicates and two technical replicates (n = 6) per each isolate ± standard deviation.

^b^Isolates were classified based on biofilm-forming ability, as described in Materials and Methods and S2 Table.

^c^Values are sialidase activity normalized to OD_600_ of the cultures expressed as the mean of three biological and two technical replicates (n = 6) per each isolate ± standard deviation. Negative values adjusted to 0.

ARDRA, amplified ribosomal DNA restriction analysis

ND, not detected

Strains of different clades were isolated from seven vaginal samples, whereas isolates from sample 058S1 harbored all three clades detected in the corresponding noncultured vaginal sample [9]. Samples 084S1 and 086S1 yielded different strains of the same clade, which was verified by random amplified polymorphic DNA (RAPD) analysis (S1 File).

ARDRA genotyping revealed that all clade 1 isolates belonged to ARDRA genotype 2 and all clade 4 isolates to ARDRA genotype 1 (Table 1). Eleven of 12 clade 2 isolates were assigned to ARDRA genotype 1, and the other isolate was assigned to ARDRA genotype 2. We did not detect ARDRA genotype 3 among the isolates, in agreement with previous results [7].

Isolates of *G*. *vaginalis* were inoculated at a standardized CFU/mL into liquid medium, and the OD_600_ was measured after 24 h of growth (Fig A in S2 File). Isolates 58.7, 84.3, and 86.5 (all clade 2) and isolates 63.1, 88.2, and 99.1 (all clade 4) demonstrated lower OD_600_ values compared with other strains. The typical growth curves were obtained for those isolates, which reached the stationary phase after 20–24 h of cultivation (Fig B in S2 File).

### VLY production by *G*. *vaginalis* subgroups

The presence of the VLY-encoding gene in the isolates was determined using a previously published PCR assay method [9]. All isolates of clades 1 and 4 were *vly* positive, whereas 5 of 12 clade 2 and 86.1 (unknown clade) isolates were *vly* negative (Table 1). To eliminate false-negative PCR results, the flanking regions of *vly* were amplified, as described previously [22]. All *vly*-negative isolates yielded a ~550-bp fragment, whereas *vly*-positive isolates produced >2500-bp fragments (S1 Fig).

**Fig 1:**
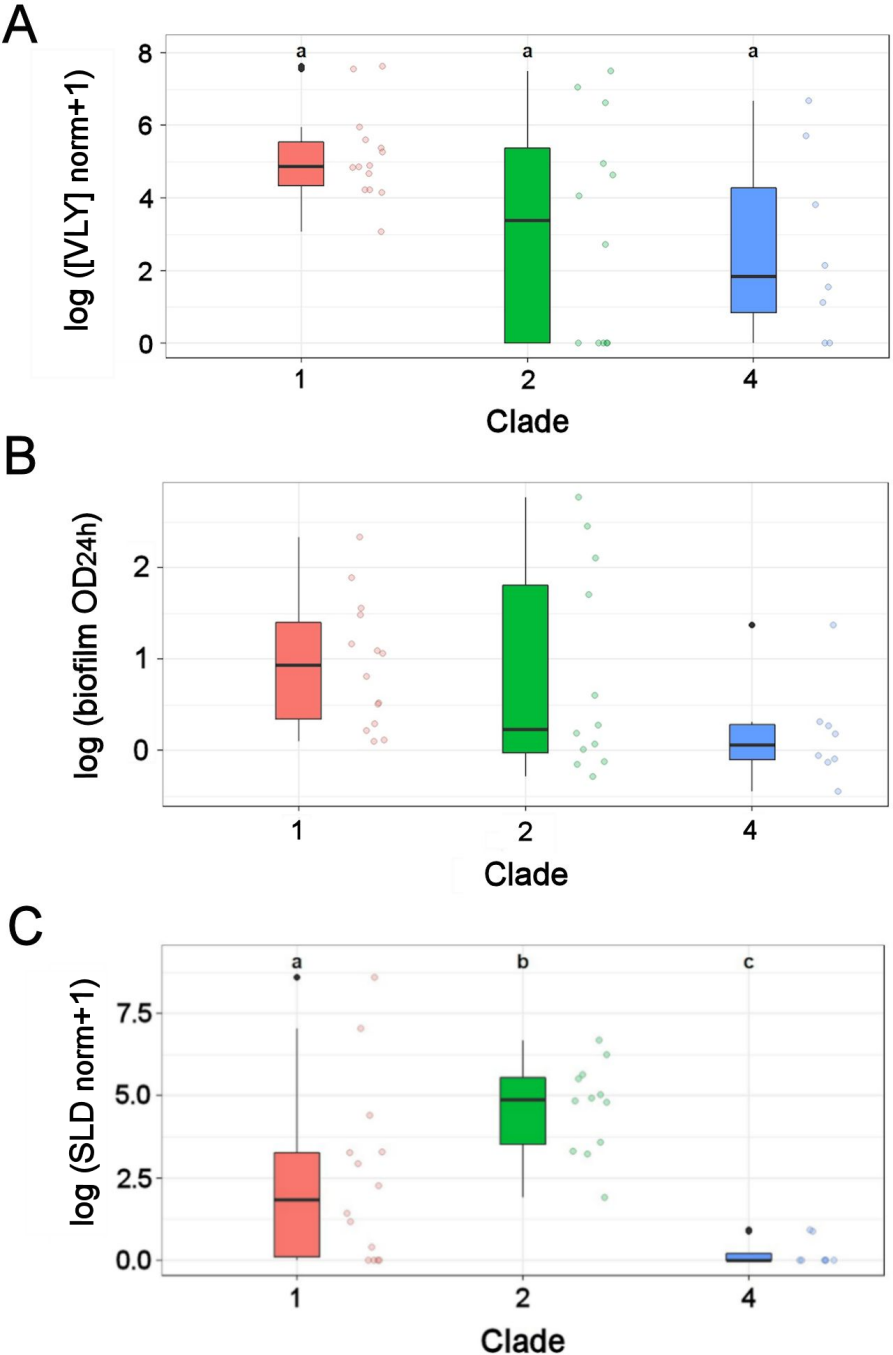
Assessment of phenotypic characteristics of *G*. *vaginalis* clinical isolates cultivated *in vitro*. (A) The ELISA-determined VLY concentration (ng/mL) was adjusted to the OD of the cultures and mapped on a log scale. (B) The OD_492_ of bacterial films after a 24-h incubation is expressed as log OD. (C) Calculated sialidase activity normalized to the OD of cultures was mapped on a log scale. Individual values are indicated by colored circles (randomly moved in the direction parallel to the X-axis to avoid overlapping) and summarized as Tukey-style boxplots [37]. Boxes restrict the interquartile range from the 25th to the 75th percentiles. Horizontal lines in the boxes indicate the median, and whiskers show the range from minimum to maximum non-outlying values. Possible outlier values are indicated by black dots. The CLD letters (where indicated) above the boxplots summarize the results of pairwise comparisons using the Games-Howell test (A) and the Conover-Iman test (C).

VLY production by *G*. *vaginalis* isolates in liquid medium was measured after 24 h of growth. The obtained VLY concentration was normalized to the OD of the bacterial cultures and compared among subgroups (Fig 1A). Overall statistically significant differences in VLY production among the clades were detected using the Welch F test (*F*(2, 14.10) = 5.00, *p* = 0.023). The subsequent *post-hoc* analysis revealed no significantly different pairs of clades (Games-Howell test, clades 1 vs. 2: *t*(14.15) = 2.16, *p* = 0.112, mean difference 2.04, 95% CI −0.42 –+4.50); clades 1 vs. 4: *t*(9.00) = 2.66, *p* = 0.062, mean difference 2.54, 95% CI −0.13 –+5.20; clades 2 vs. 4: *t*(16.98) = 0.40, *p* = 0.916, mean difference 0.50, 95% CI −2.72 –+3.72).

### Biofilm-forming ability of *G*. *vaginalis* subgroups

All isolates were cultured in BHIs medium in 96-well microplates for 24 and 48 h to evaluate biofilm formation *in vitro*. The amount of biofilm produced was quantified by safranin staining. Prior reports indicate that incubation period can affect biofilm accumulation by certain *G*. *vaginalis* strains [38]. Our data showed that differences among isolates in biofilm amount after 24 h and 48 h incubation were not statistically significant (paired samples *t*-test, *t*(35) = 0.057, *p* = 0.955). Comparing the amount of biofilm by clades, no statistically significant differences were detected either after a 24-h (Kruskal-Wallis test, *H*(2) = 6.50, *p* = 0.059) or 48-h (*H*(2) = 3.34, *p* = 0.188) incubation (Fig 1B). To reveal biofilm-forming ability of individual *G*. *vaginalis* isolates, they were classified into four categories (Table 1, S2 Table), as described previously [24].

### Sialidase activity in *G*. *vaginalis* subgroups

The presence of the sialidase gene was determined by PCR using two sets of primers, as described in [9]. All clade 1 and 2 isolates and unknown clade strain 86.1 were *sld* positive, whereas all clade 4 isolates were *sld* negative. It was demonstrated that a portion of *G*. *vaginalis* sialidase activity was cell-associated, but a fraction of the activity remained in the bacterial supernatant [11]. Quantitative assay of sialidase activity in *G*. *vaginalis* culture supernatants using the fluorogenic substrate MUN showed that 5 of 14 clade 1 isolates were activity negative even though they were *sld* positive. All clade 2 isolates were activity positive (Table 1). The analysis of sialidase activity indicated statistically significant differences Kruskal-Wallis test, *H*(2) = 17.60, *p* < 0.001) (Fig 1C). The subsequent *post hoc* analysis using the Conover-Iman test confirmed that all three clades differed significantly (clade 1 vs. 2: *t* = 3.37, *p* = 0.004; clade 1 vs. 4: *t* = 2.92, *p* = 0.006; clade 2 vs. 4: *t* = 5.74, *p* < 0.001). The data presented in Fig 1C demonstrate that clade 2 had the highest, clade 1 intermediate, and clade 4 the lowest sialidase activity.

### Activity of *G*. *vaginalis* recombinant sialidase

**Fig 2:**
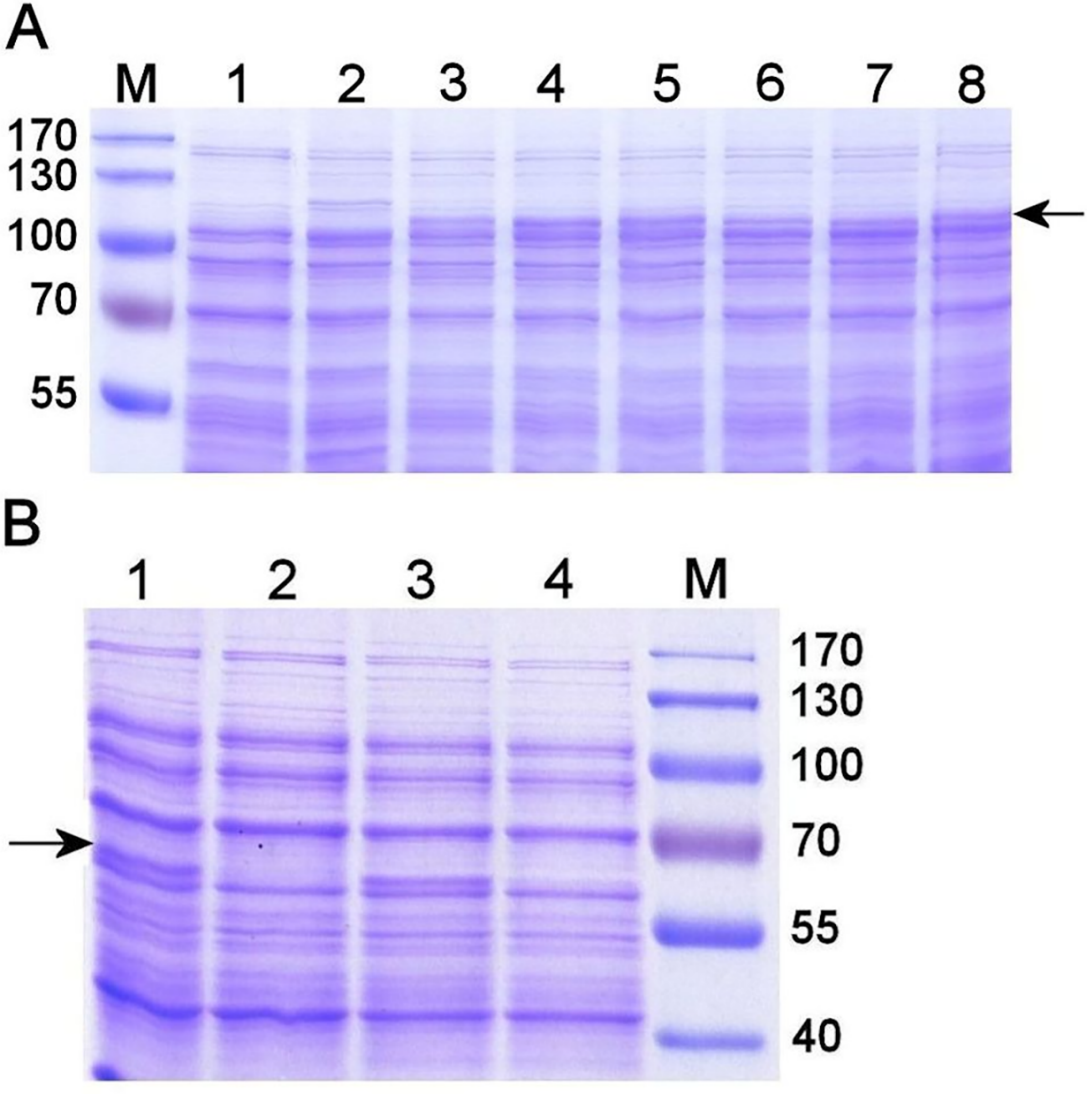
SDS-PAGE analysis of recombinant sialidases in *E*. *coli* lysates. (A) Filtered soluble fraction of cell lysates containing full-length sialidase from *G*. *vaginalis* isolates 47.3 (lane 3), 58.4 (lane 4), 60.1 (lane 5), 79.2 (lane 6), 86.1 (lane 7), and 114.2 (lane 8). As controls, filtered soluble fractions of *E*. *coli* cell lysate before induction (lane 1) and of non-transformed cells (lane 2) were loaded on the gel. (B) The catalytic domain of sialidase from *G*. *vaginalis* isolates 86.1 and 114.2 was expressed in *E*. *coli*, and the cleared cell lysates before (lanes 1 and 3) and after (lanes 2 and 4) filtration were analyzed. M, Page Ruler Prestained Protein Ladder (Thermo Fisher Scientific). Arrows indicate the migration positions of recombinant sialidases.

The *G*. *vaginalis* putative sialidase gene encodes a protein of 907 amino acids [7, 11, 19] with a predicted molecular mass of 99.8 kDa. The DNA fragment corresponding the full-length *sld* gene was amplified using genomic DNA extracted from *G*. *vaginalis* isolates 47.3 and 58.4 that exhibited high sialidase activity in the culture medium supernatant and sialidase activity–negative isolates 60.1, 79.2, 86.1, and 114.2 (Table 1). Isolates 60.1 and 114.2 had very low sialidase activity (Table 1); therefore, we classified them as sialidase negative [11]. The boundaries of the catalytic non-viral sialidase domain spanned amino acids 391–844 of the reference Genbank Protein Accession sequence YP_003985295 (*G*. *vaginalis* ATCC 14019), as determined by a search of the NCBI Conserved Domain Database [39]. The fragments comprised the putative catalytic domain corresponding to amino acids 374–907 of the reference sequence. Amplification was performed using template DNA extracted from isolates 86.1 and 114.2. The nucleotide sequences of the full-length sialidase genes were deposited in GenBank under accession numbers MG737371–MG737376. The recombinant full-length and truncated proteins were expressed as an N-terminal His-tagged form in *E*. *coli* and detected in the total cell lysate on SDS-PAGE as bands with a molecular mass of ~110 kDa and ~65 kDa, respectively, in agreement with the calculated molecular mass of sialidase. The full-length proteins were found in the soluble fraction of the cell lysate and remained in solution after filtration (Fig 2A). The truncated proteins were poorly soluble and not detected on the gel after filtration (Fig 2B).

**Fig 3:**
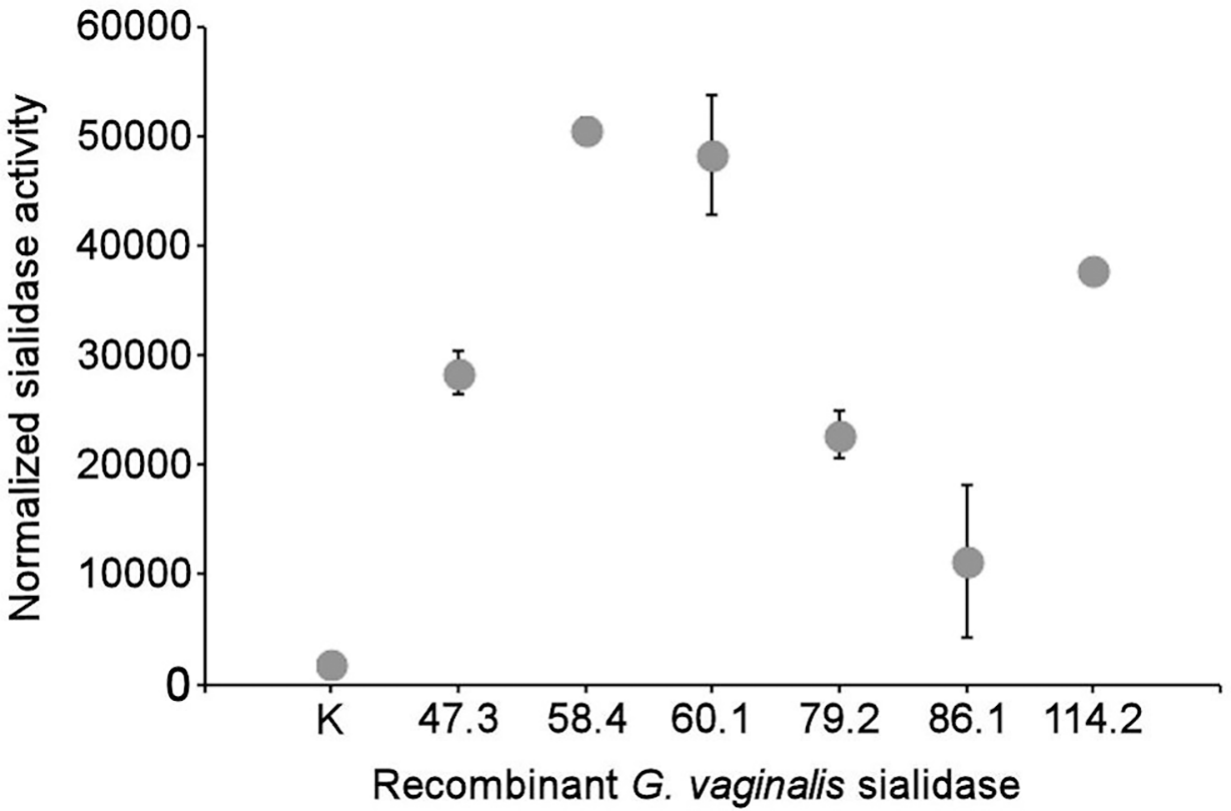
Activity of recombinant *G*. *vaginalis* sialidases in *E*. *coli* culture supernatants. The full-length *sld* gene from *G*. *vaginalis* isolates 47.3, 58.4, 60.1, 79.2, 86.1, and 114.2 was expressed in *E*. *coli*, and sialidase activity was assessed in the soluble fraction of the cell lysate. Sialidase activity was normalized to the total protein in the sample. K, soluble fraction of non-transformed *E*. *coli* cells (negative control). Error bars represent SD.

Fractions of *E*. *coli* cells containing full-length recombinant sialidase were screened for enzymatic activity using the fluorogenic substrate MUN. The resulting quantitative sialidase activity data were normalized to total protein content in the soluble cell fraction. The samples containing recombinant sialidases from both sialidase activity–positive and–negative isolates exhibited enzymatic activity (Fig 3). Control non-transformed *E*. *coli* cells subjected to the same treatment as transformed cells did not show sialidase activity. Multiple alignment of the catalytic domain sequences of sialidase activity–positive and–negative *G*. *vaginalis* isolates (S3 File) suggested the presence of all conserved motifs characteristic of the catalytic domain of sialidases [40, 41].

### Correlation analysis of phenotypic characteristics of *G*. *vaginalis* isolates

Spearman’s rank correlation analysis was performed to determine the relationship between the following phenotypic characteristics of the isolates: VLY production, biofilm amount after 24 h and 48 h of incubation, and sialidase activity. Biofilm amount (24 h and 48 h) and VLY amount were statistically significantly correlated (*p* < 0.05, Table 2). Sialidase activity was not statistically significantly correlated with the other properties (*p* ≥ 0.05, Table 2).

**Table 2:** The results of Spearman’s correlation analysis.

Analyzed variables	r_s_^c^	95% CI_lower_	95% CI_upper_	*p*
Biofilm 24 h^a^ vs. 48 h^b^	0.75	0.47	0.90	<0.001 *
Biofilm 24 h vs. VLY production	0.50	0.12	0.72	0.010 *
Biofilm 48 h vs. VLY production	0.43	0.02	0.69	0.039 *
Biofilm 48 h vs. sialidase activity	0.13	-0.26	0.47	>0.999
Biofilm 24 h vs. sialidase activity	0.12	-0.22	0.42	>0.999
Sialidase activity vs. VLY production	0.10	-0.30	0.48	>0.999

The rows of the table are arranged in descending order of Spearman’s correlation coefficients.

^a^Biofilm amount as measured using OD_492_ after 24-h incubation.

^b^Biofilm amount as measured using OD_492_ after 48-h incubation.

^c^Spearman's rank-order correlation coefficient.

*Statistically significant correlation.

### Classification analysis of phenotypic characteristics of *G*. *vaginalis* isolates

**Fig 4:**
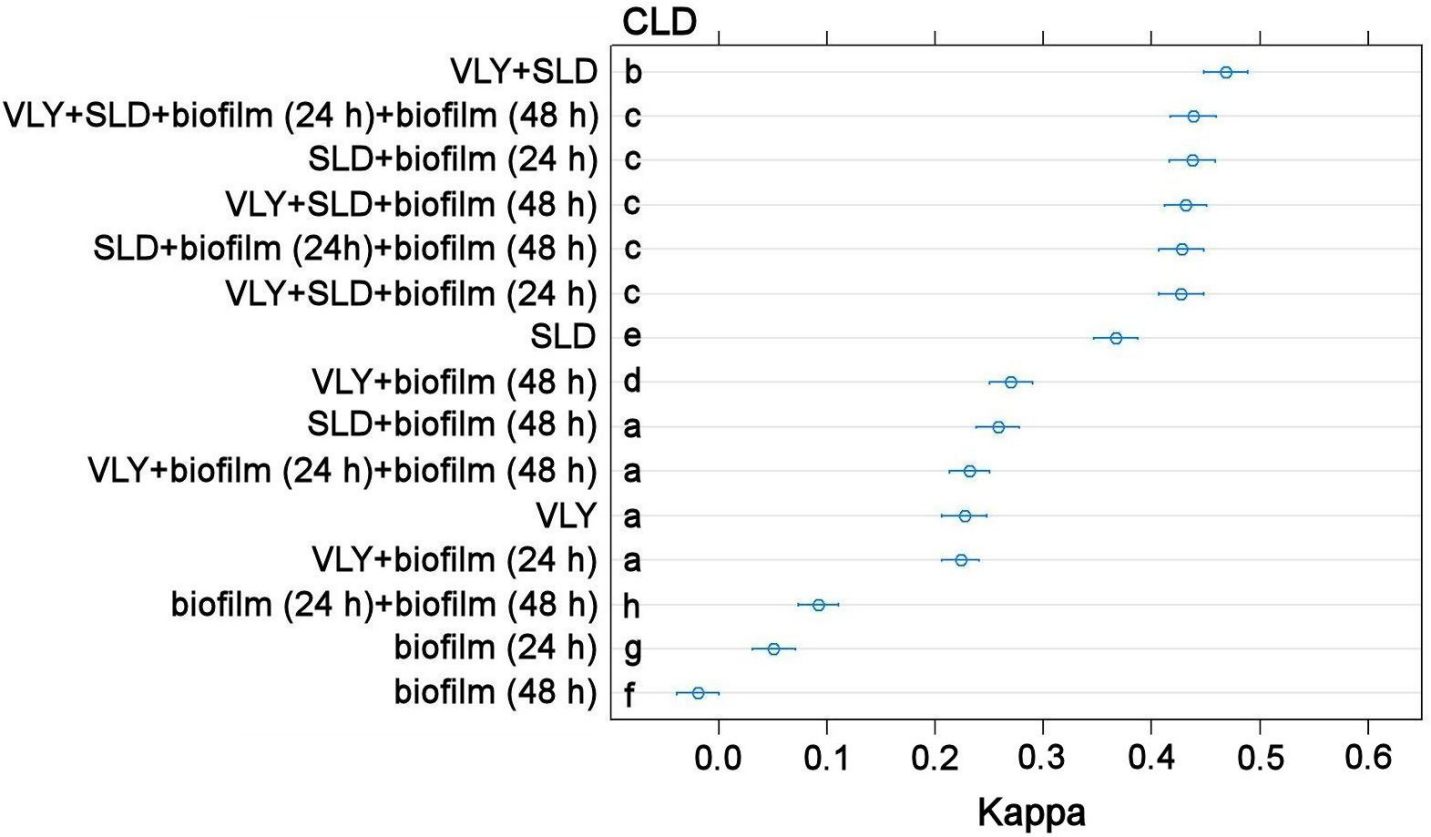
Classification accuracy of numerical features expressed by Cohen kappa values. The models were sorted in descending order according to mean kappa value. Blue circles represent mean kappa values. Error bars indicate 95% CI. VLY, vaginolysin amount; SLD, sialidase activity; biofilm (24 h), biofilm amount after 24 h incubation; biofilm (48 h), biofilm amount after 48 h incubation. The results of pairwise comparisons of each model’s performance using the paired *t*-test are summarized using the compact letter display (CLD) method.

The four phenotypic characteristics were investigated as predictors to identify *G*. *vaginalis* subgroups via a random forest algorithm. Classification performance for single predictor models was evaluated as follows: sialidase activity as a predictor resulted in kappa = 0.37 (95% CI 0.35–0.39), VLY amount as a predictor gave kappa = 0.23 (95% CI 0.21–0.25), biofilm amount with 24 h incubation gave kappa = 0.05 (95% CI 0.03–0.07), and biofilm amount with 48 h incubation gave kappa = –0.02 (95% CI −0.04–0.00). The differences between all pairs of the four kappa values were statistically significant (paired *t*-test, *p* < 0.05). All models exhibiting the best performance included sialidase activity as a predictor (see top rows in Fig 4). The highest kappa value (0.47, 95% CI 0.45–0.49) was obtained using a combination of VLY amount and sialidase activity as predictors. The mean kappa value of this combination was statistically significantly different from the other combinations, as it does not share any common CLD letter in Fig 4 (paired *t*-test, *p* < 0.05 in all cases with no shared CLD letters). The results of the classification analysis are summarized in Fig 4.

### Cluster analysis of *G*. *vaginalis* isolates

**Fig 5:**
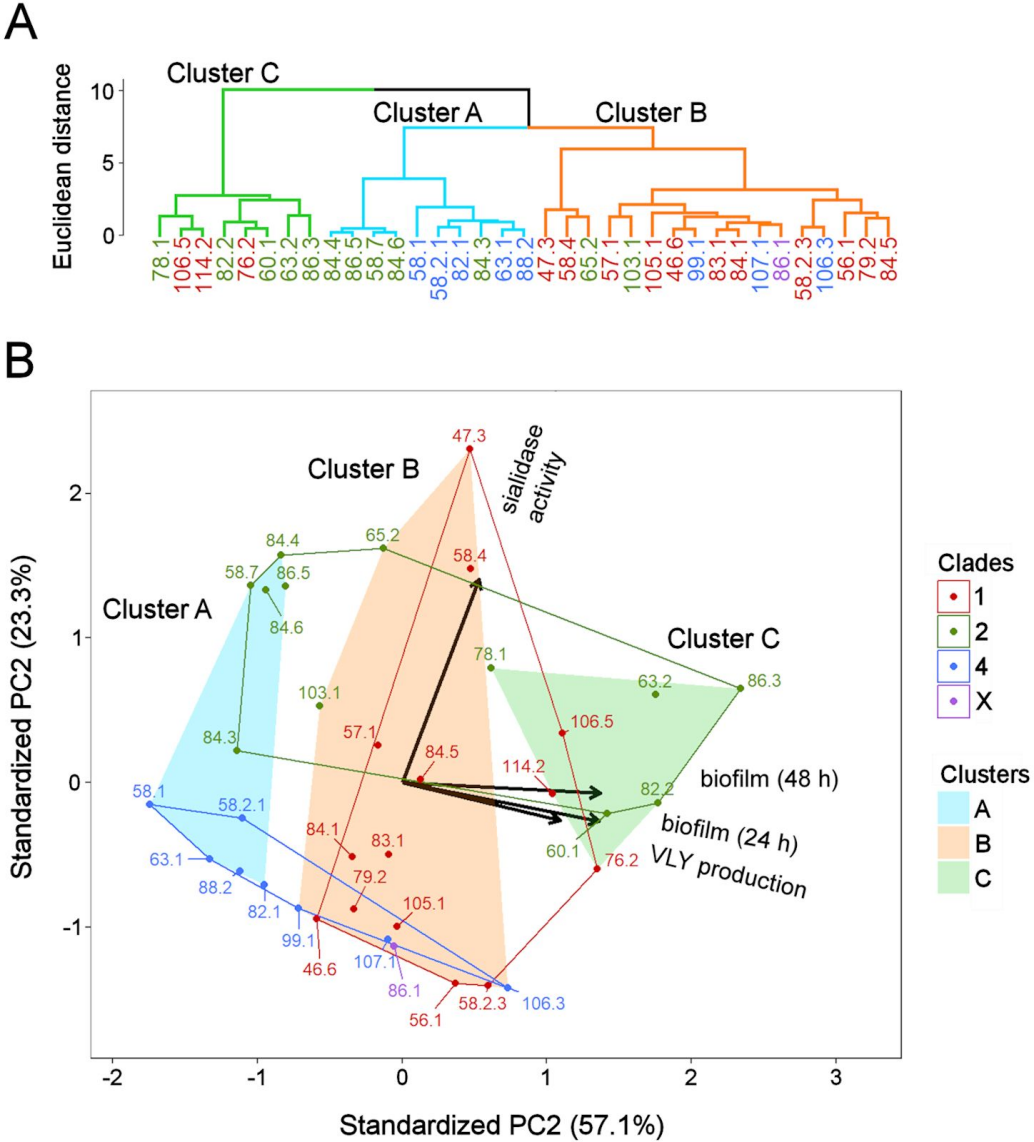
Results of hierarchical clustering and principal component analysis based on four characteristics: Sialidase activity, VLY production, and biofilm amount after 24 h and 48 h incubation. (A) Dendogram. The colors of the branches represent the three largest clusters. (B) PCA biplot. Axes represent the first and the second principal components (PCs). Percentages on the axes indicate the percentage of total variance explained by each PC. Arrows in the biplot represent the phenotypic characteristics. Dots indicate isolates, and the colors of the dots/isolate numbers correspond to particular clades. Closed lines indicate the areas in the space of PCs restricted by the isolates of the same clade. Shaded fields indicate areas restricted by the clusters determined in the dendogram.

The results of the classification analysis of phenotypic properties only partially reflected the subgrouping of *G*. *vaginalis* isolates; therefore, an exploratory analysis was performed via agglomerative hierarchical clustering (HC) and principal component analysis (PCA). The first HC and PCA was based on all four phenotypic (numeric) characteristics (Fig 5). The first two principal components (PCs) accounted for 80.4% of the total variance, suggesting that the model was a good representation of the data structure according to a biplot (Fig 5B). The arrangement of the original clades in the space of the PCs demonstrated that the areas occupied by the most dissimilar clades (clades 2 and 4) did not overlap (Fig 5). This was determined primarily based on sialidase activity and was in agreement with the results of the classification analysis. The area of clade 1 highly overlapped with the areas of clades 2 and 4.

The three correlated characteristics, namely, biofilm amount after 24 h and 48 h incubation and VLY production, were represented by arrows in the biplot and pointed in almost the same direction (Fig 5B). The low angles between these arrows suggest that the features convey similar information. Moreover, it can be interpreted that the structures of the clusters (shaded areas) were determined by these three characteristics, as the bonds of the clusters were situated nearly perpendicular to the arrows. By contrast, the almost perpendicular arrow depicting sialidase activity suggests that this feature conveys more diverse information than biofilm or VLY production, and it had little influence on cluster structure (Fig 5B).

**Fig 6:**
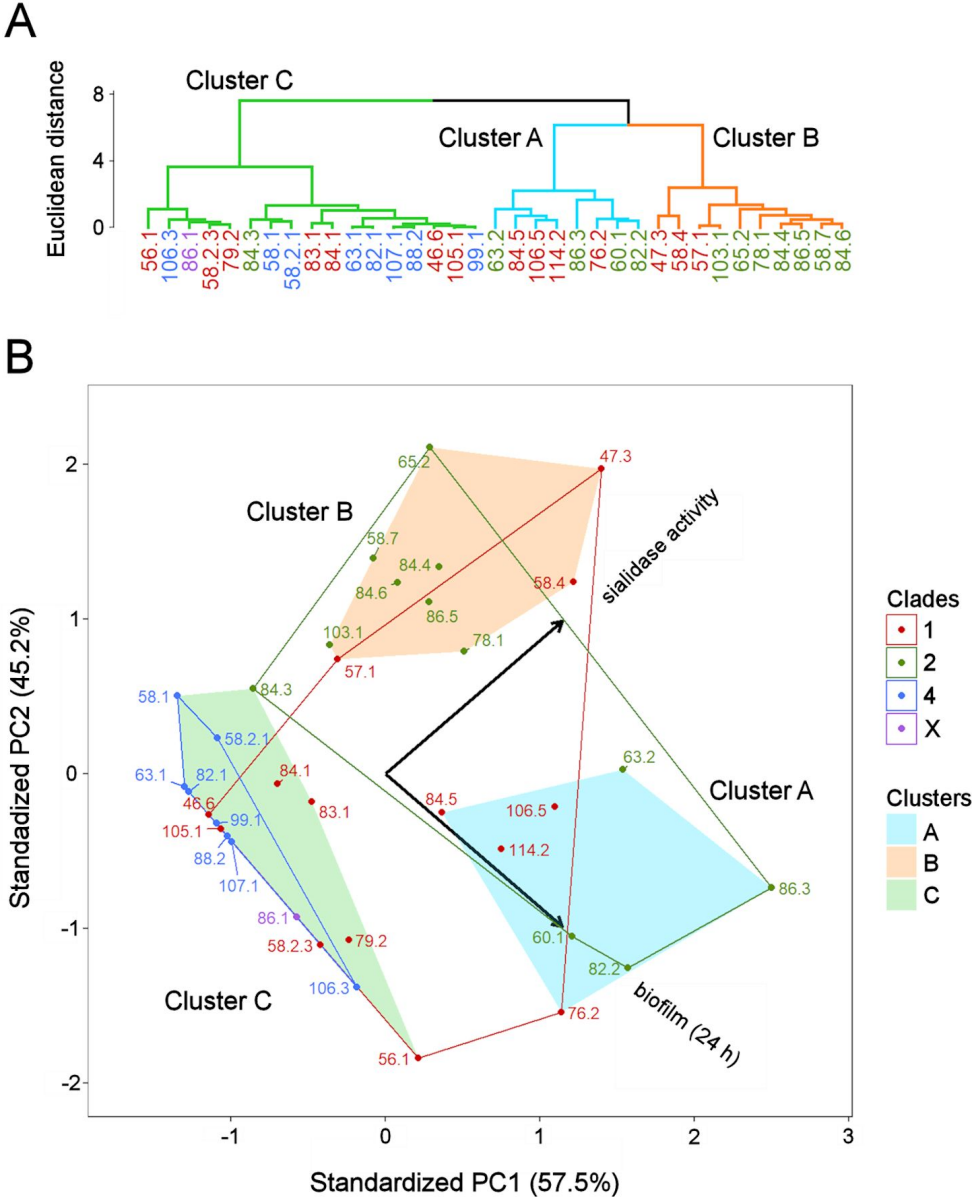
Results of hierarchical clustering and principal component analysis based on two characteristics: Sialidase activity and biofilm amount after 24 h incubation. (A) Dendogram that shows similarity between the *G*. *vaginalis* isolates. The colors of the branches represent the three largest clusters. (B) PCA biplot. Axes represent the first and the second principal components (PCs). Percentages on the axes indicate the percentage of the total variance explained by each PC. Arrows in the biplot represent the phenotypic characteristics. Dots indicate isolates, and the colors of the dots/isolate numbers correspond to particular clades. Closed lines indicate the areas in the space of PCs restricted by the isolates of the same clade. Shaded fields indicate areas restricted by the clusters determined in the dendogram.

It could be speculated that the impact of the information conveyed by the three correlated characteristics is overestimated compared with the information represented by sialidase activity; thus, the resulting clustering is not optimal. In order to equalize the impacts of the two sources of information, two of three correlated properties were excluded and the property that in combination with sialidase activity resulted in the highest value of Calinski-Harabasz (CH) index was selected. Thus, the final clustering was based on sialidase activity and biofilm amount after 24 h incubation (CH = 57.1) (Fig 6). The other combinations based on biofilm amount after 48 h incubation + sialidase activity (CH = 51.9) or VLY production + sialidase activity (CH = 30.8) were rejected.

Analysis of the relative positions of the clusters with respect to the directions of the arrows in Fig 6 demonstrated that cluster C is characterized by lower sialidase activity than clusters A and B. Moreover, cluster B is characterized by lower biofilm amount than cluster A. All clade 4 isolates were found in cluster C, whereas the isolates of clade 1 were distributed throughout all clusters. All isolates of clade 2 belong to clusters A and B, except isolate 84.3, which belongs to cluster C.

## Discussion

In this study, we analyzed *G*. *vaginalis* clinical isolates of three subgroups (clades 1, 2, and 4) with respect to their ability to produce the toxin VLY, form a biofilm, and secrete active sialidase. VLY, a virulence factor produced by *G*. *vaginalis*, is a cholesterol-dependent cytolysin, the activity of which is potentiated by human complement glycoprotein CD59 [12, 13]. Although there is no direct evidence describing the role of VLY *in vivo*, researchers have suggested that high levels of expression of this cytolysin are associated with high cytotoxicity [20, 42, 43]. *Gardnerella vaginalis* clade 2 was the most variable with respect to the presence of the *vly* gene. Moreover, we found that a single vaginal sample (e.g., 086S1) could yield clade 2 isolates with and without the *vly* gene. Although, statistically significant differences in VLY production among the clades were detected, the subsequent *post-hoc* analysis revealed no significantly different pairs of clades. In respect of VLY amount, the relatively high strain-to-strain variability among isolates of clades 2 and 4 was observed, whereas clade 1 was more homogeneous.

Desquamated vaginal epithelial cells covered with a polymicrobial bacterial biofilm composed mainly of *G*. *vaginalis* were detected in BV patients [16, 17]. Moreover, biofilm formation is associated with increased antimicrobial resistance and the recurrence of disease, and creates the possibility for transmission through sexual contact [17, 44]. Despite limitations of the *in vitro* biofilm-forming assay, the potential for isolates to express biofilm-associated genes was demonstrated in the current study. Clade 1 exhibited greater internal variability with respect to biofilm amount, whereas the vast majority of clade 4 isolates formed either little or no biofilm. Nevertheless, differences in biofilm amount among the clades did not reach statistical significance.

Elevated activities of sialidases and other glycosidases were detected in vaginal fluids of BV patients [14, 15, 45]. Recent studies using *in vivo* and *in vitro* models showed that *G*. *vaginalis* sialidase promotes depletion of mucus and the degradation of secretory immunoglobulin A [11, 45–47]. Despite the presence of the *sld* gene, some *G*. *vaginalis* isolates test negative for sialidase activity [7, 9, 11, 19]. In this study, detection of *sld* and quantification of sialidase activity confirmed previous findings that clade 4 (subgroup A) isolates are negative for sialidase activity [7]. Statistically significant differences in sialidase activity were found among the three subgroups. On average, clade 2 had an approximately 2-fold higher sialidase activity than the other two clades, in agreement with previous results [7]. Statistical classification analysis revealed that in comparison to other analyzed phenotypic characteristics, sialidase activity has best predictive properties for identifying the clades.

Kinetic analysis of MUN hydrolysis using crude extracts of *E*. *coli* expressing *G*. *vaginalis* sialidases confirmed that enzymatic activity is the result of the translated product of a gene previously designated as a putative sialidase gene [7, 9, 11, 18]. Supernatants of *G*. *vaginalis sld*-negative isolates did not show sialidase activity, suggesting the absence of an alternative gene encoding this enzyme. This result should be confirmed using the qualitative filter spot test, which also detects cell-associated sialidase. We also showed that recombinant *G*. *vaginalis* sialidases from both sialidase activity–positive and–negative isolates demonstrated enzymatic activity. This suggests that regulation of the *sld* gene at the transcription level likely determines sialidase inactivity in cultured *G*. *vaginalis*.

Analysis of virulence factors raised the question as to whether the phenotypic characteristics could define the subgroups of *G*. *vaginalis*. Comparison of single-predictor models in the classification analysis showed that the three clades could be best identified based on sialidase activity (kappa = 0.37). The next best feature was VLY production (kappa = 0.23). Predictive power of biofilm amount after 24 h incubation was extremely limited (kappa = 0.05). Biofilm amount after 48 h incubation had no predictive power (kappa = -0.02). A combination of sialidase activity and VLY production significantly increased the classification performance (kappa = 0.47). This could be associated with a low insignificant (*r_s_* = 0.10, *p* > 0.999) correlation between sialidase activity and VLY production suggesting that these two characteristics convey different information. Thus, the *G*. *vaginalis* clades could be best defined based on the profiles of two phenotypic characteristics: sialidase activity and VLY production. These profiles allowed correct identification of clades only in a fraction of *G*. *vaginalis* isolates, suggesting that there is room for exploring how the subgroups could be defined more properly.

Further statistical exploratory data analysis of phenotypic characteristics assayed *in vitro* by means of HC and PCA revealed that clades 2 and 4 are the most dissimilar subgroups. Furthermore, it showed that clade 4 is the most homogenous subgroup: all isolates of this clade were found in the same cluster, which was characterized by low production levels of all studied virulence factors. By contrast, both clades 1 and 2 exhibited greater internal variability; clade 2 isolates were mainly distributed between two clusters, whereas clade 1 isolates were found in all three clusters and characterized by a distinct profile of phenotypic characteristics.

Clade 3 was the less prevalent clade in vaginal samples of Lithuanian women [9]. This could be one of the reasons why we were not able to isolate the strains of clade 3 and evaluate their virulence potential. In other study, the isolates of subgroup D (clade 3) from Kenya and Canada were obtained and analysed [7], which demonstrates the potential of the clade-based approach for *G*. *vaginalis* subtyping. Clade 3 isolates required for further investigation.

The identified differences in the virulence potential of *G*. *vaginalis* subgroups might be clinically relevant as previous studies demonstrated a positive association of certain clades with BV [9, 21, 48]. In particular, clade 1 was positively associated with a high Nugent score (NS), whereas clade 4 showed no association with BV [9, 21, 48]. Clade 2 was significantly more common in BV-positive (NS 7–10) samples from Lithuania [9], whereas that clade was previously reported to be associated with intermediate vaginal flora (NS 4–6) in samples from the USA [21]. Clade 3 demonstrated no association with the disorder [9], whereas Balashov and colleagues [21] found a positive association between clade 3 and BV.

In our study the majority of the isolates were derived from vaginal samples with NS ≥ 4, therefore characterization of isolates of various subgroups from the BV-negative women would help to elucidate the role of *G*. *vaginalis* in health and disease. Our findings propose that *G*. *vaginalis* subgroups might play distinct roles in vaginal microbiota. The studies on the abundance of *G*. *vaginalis* clades in various vaginal conditions [48] are of particular importance.

## Conclusions

In this study, we identified phenotypic characteristics associated with the virulence of three subgroups (clades) of *G*. *vaginalis*. We found that only sialidase activity, an important virulence factor, is differentially distributed among subgroups. We also showed that sialidase activity is linked with a gene previously designated as the putative *sld* gene. The fact that some isolates containing the sialidase gene do not exhibit the enzymatic activity may be explained by differential regulation of *sld* at the transcriptional level. Subgrouping of all isolates by means of statistical analysis into clusters clearly demonstrated that clade 4 exhibits low virulence potential. However, the internal diversity of both clades 1 and 2 in terms of production of virulence factors *in vitro* suggests the need for further studies with more isolates, including analyses of other significant virulence, metabolic, and immunomodulatory characteristics of *G*. *vaginalis*.

## Supporting information

S1 Table Primers used in this study. (PDF)

S2 Table Classification of *G*. *vaginalis* isolates by biofilm-forming ability. (PDF)

S1 File Random amplified polymorphic DNA (RAPD) analysis of *G*. *vaginalis* isolates. (PDF)

S2 File **(A) Optical density (OD**_**600**_**) of *G*. *vaginalis* isolates after 24 h incubation.** The growth assay of each isolate in liquid medium was performed as three independent experiments in duplicate. Error bars indicate standard deviation (SD). **(B) The growth curves of isolates 58.7, 63.1, 84.3, 86.5, 88.2, and 99.1.** Isolate 58.2.1was used as a control. The growth curves were generated from two independent experiments in duplicate as described previously [19]. Error bars indicate standard deviation (SD).
(PDF)

S3 File Alignment of amino acid sequences of *G*. *vaginalis* sialidase catalytic domain. (PDF)

S1 Fig Amplification of *vly* gene using primers flanking its region. *G*. *vaginalis* isolates 84.5, 86.3, and 88.2 were *vly* positive, whereas isolates 58.7, 84.3, 84.4, 84.6, 86.1, and 86.5 were *vly* negative. M, Gene Ruler DNA Ladder Mix (Thermo Fisher Scientific). The PCR results were verified by the ELISA quantification of VLY in bacterial supernatants.
(PDF)
