# Supplementary material for: Phenotypic characterization of Gardnerella vaginalis subgroups suggests differences in their virulence potential
Source: PLoS One. 2018 Jul 12;13(7):e0200625. doi: 10.1371/journal.pone.0200625 (PMC6042761; doi:10.1371/journal.pone.0200625)
Supplement: S1 Table — (PDF) [file pone.0200625.s001.pdf]

S1 Table. Primers used in this study.

Target	Primer	Amplicon size, bp	Primer sequence (5'-3')	Reference
Putative alfa-L-fucosidase	GV1-fuc1-S	139	CCAGTCATAAGTTTGCGTTTTACC	[21]
	GV1-fuc1-AS		TGGCACTGGCAAAGTTTACAAC	
Hypothetical protein	GV2-hyp-S	124	GCAAAGCAGACTGAGCGTATTAG	[21]
	GV2-hyp-AS		GTAATAATCAGGCTCCTCATCGC	
Thioredoxin	GV3-thi-S	142	TTCTGCTTCTTCTGCTATTTGCTG	[21]
	GV3-thi-AS		TTCGTTGACTTTTGGGCAACATG	
Chloride transporter	GV4-cis-S	74	CCTACGCAAGCTCCAGACGAC	[21]
	GV4-cis-AS		ACAAGTTGCACTCTTCGAGCTGG	
16S rRNA	Fwd-Gar	1434	TTCGATTCTGGCTCAGG	[4]
	Rev-Gar		CCATCCCAAAAGGGTTAGGC	
Vaginolysin	VLY-585F	749	GTACGATTCTGCAAGCGCACAAAGC	[9]
	VLY-1334R		CCTTCCCAAGCGCGAGAACGC	
vly flanking region	VLY-FL-F		CATCTTCGCCAGCAACTTCC	[22]
	VLY-FL-R		GGCGGAATTATGTGCGTTATTGG	
sld gene	Sia1F	704	ATGGAACGTCGTTCAACGAAG	[9]
	Sia1R		GATACGCGTTTTATGTCTCTTGC	
sld gene	Sia2F	988	CACGTGGAACATATGGAAATCG	[9]
	Sia3R		AAATGTCTCTTCCATGTTGGCT	
Full-length *sld* gene	Sia5-F	~2700	AACACTAGTATGGAACGTCGTTCAAC	This study
	Sia3-R		ATTGTCGACTTAAATGTCTCTTCCATKTTG	
Catalytic domain of *sld* gene	SiaK-F	~1600	AACACTAGTCTAACCGAAGGMCAAATC	This study
	Sia3-R		ATTGTCGACTTAAATGTCTCTTCCATKTTG	
