# Supplementary material for: Phenotypic characterization of Gardnerella vaginalis subgroups suggests differences in their virulence potential
Source: PLoS One. 2018 Jul 12;13(7):e0200625. doi: 10.1371/journal.pone.0200625 (PMC6042761; doi:10.1371/journal.pone.0200625)
Supplement: S2 Table — (PDF) [file pone.0200625.s002.pdf]

S2 Table. Classification of *G. vaginalis* isolates by biofilm-forming ability

Isolate	Clade	24 h incubation						48 h incubation					
		Mean OD[a]	ODc[b]	2xODc	4xODc	OD biofilm[c]	Biofilm former[d]	Mean OD[a]	ODc[b]	2xODc	4xODc	OD biofilm[c]	Biofilm former[d]
46.6	1	0.047 ± 0.004	0.061	0.121	0.243	0.068± 0.005	weak	0.059 ± 0.007	0.079	0.157	0.314	0.075± 0.003	non
47.3	1	0.090 ± 0.006	0.109	0.219	0.438	0.184 ± 0.010	weak	0.047 ± 0.004	0.061	0.121	0.243	0.146 ± 0.015	moderate
56.1	1	0.064 ± 0.004	0.077	0.153	0.306	0.507 ± 0.043	strong	0.058 ± 0.012	0.095	0.191	0.381	0.106 ± 0.007	weak
57.1	1	0.088 ± 0.006	0.107	0.214	0.429	0.118 ± 0.007	weak	0.047 ± 0.004	0.061	0.121	0.243	0.074 ± 0.009	weak
58.1	4	0.057 ± 0.022	0.124	0.248	0.496	0.079 ± 0.017	non	0.050 ± 0.013	0.090	0.180	0.360	0.067 ± 0.002	non
58.4	1	0.040± 0.003	0.046	0.093	0.185	0.104 ± 0.005	moderate	0.059 ± 0.028	0.143	0.286	0.572	0.130 ± 0.013	non
58.7	2	0.076 ± 0.012	0.111	0.223	0.446	0.098 ± 0.011	non	0.056 ± 0.010	0.086	0.172	0.343	0.078 ± 0.008	non
58S2.1	4	0.061 ± 0.011	0.095	0.189	0.379	0.083 ± 0.005	non	0.059 ± 0.005	0.074	0.149	0.298	0.075 ± 0.003	weak
58S2.3	1	0.058 ± 0.004	0.072	0.143	0.286	0.208 ± 0.011	moderate	0.060 ± 0.001	0.064	0.128	0.257	0.175 ± 0.012	moderate
60.1	2	0.059 ± 0.008	0.084	0.169	0.338	0.691 ± 0.031	strong	0.063 ± 0.004	0.075	0.151	0.302	0.830 ± 0.056	strong
63.1	4	0.061 ± 0.005	0.076	0.152	0.303	0.069 ± 0.007	non	0.063 ± 0.004	0.075	0.150	0.300	0.067 ± 0.004	non
63.2	2	0.055 ± 0.014	0.097	0.195	0.390	0.537 ± 0.046	strong	0.063 ± 0.015	0.107	0.214	0.428	1.187 ± 0.197	strong
65.2	2	0.052 ± 0.014	0.094	0.188	0.376	0.070 ± 0.009	non	0.061 ± 0.015	0.105	0.210	0.420	0.091 ± 0.008	non
76.2	1	0.056 ± 0.014	0.098	0.196	0.392	1.009 ± 0.082	strong	0.053 ± 0.014	0.094	0.187	0.374	0.897 ± 0.048	strong
78.1	2	0.045 ± 0.005	0.060	0.119	0.239	0.109 ± 0.035	weak	0.063 ± 0.003	0.073	0.146	0.292	0.406 ± 0.036	strong
79.2	1	0.070 ± 0.012	0.106	0.213	0.426	0.342 ± 0.047	moderate	0.069 ± 0.003	0.077	0.154	0.308	0.070 ± 0.01	non
82.1	4	0.066 ± 0.003	0.075	0.150	0.299	0.071 ± 0.007	non	0.058 ± 0.004	0.070	0.141	0.282	0.080 ± 0.008	weak
82.2	2	0.059 ± 0.018	0.112	0.224	0.448	1.299 ± 0.10	strong	0.058 ± 0.004	0.069	0.139	0.278	1.453 ± 0.061	strong

83.1	1	0.049 ± 0.005	0.063	0.127	0.254	0.105 ± 0.009	weak	0.064 ± 0.006	0.082	0.164	0.328	0.146 ± 0.013	weak
84.1	1	0.053 ± 0.011	0.085	0.170	0.340	0.114 ± 0.048	weak	0.048 ± 0.014	0.089	0.177	0.355	0.117 ± 0.053	weak
84.3	2	0.058 ± 0.005	0.074	0.148	0.296	0.063 ± 0.010	non	0.062 ± 0.004	0.07	0.149	0.297	0.087 ± 0.007	weak
84.4	2	0.052 ± 0.001	0.054	0.107	0.214	0.065 ± 0.016	weak	0.061 ± 0.004	0.072	0.144	0.289	0.070 ± 0.009	non
84.5	1	0.058± 0.006	0.074	0.149	0.297	0.222 ± 0.032	moderate	0.059 ± 0.004	0.072	0.144	0.287	0.109 ± 0.013	weak
84.6	2	0.054 ± 0.003	0.062	0.123	0.246	0.066 ± 0.021	weak	0.058 ± 0.003	0.067	0.134	0.268	0.063 ± 0.007	non
86.1	ND	0.058 ± 0.006	0.075	0.149	0.299	0.177 ± 0.082	moderate	0.060 ± 0.002	0.067	0.134	0.269	0.106 ± 0.014	weak
86.3	2	0.058 ± 0.001	0.063	0.126	0.252	1.006 ± 0.335	strong	0.062 ± 0.002	0.068	0.136	0.272	1.198 ± 0.103	strong
86.5	2	0.054 ± 0.003	0.062	0.123	0.246	0.081 ± 0.007	weak	0.060 ± 0.005	0.074	0.149	0.298	0.076 ± 0.006	weak
88.2	4	0.059 ± 0.006	0.078	0.155	0.310	0.102 ± 0.006	weak	0.060 ± 0.005	0.074	0.149	0.298	0.075 ± 0.019	weak
99.1	4	0.059 ± 0.001	0.062	0.123	0.247	0.074 ± 0.016	weak	0.060 ± 0.005	0.076	0.151	0.303	0.069 ± 0.020	non
103.1	2	0.056 ± 0.008	0.080	0.160	0.321	0.081 ± 0.005	weak	0.056 ± 0.001	0.060	0.120	0.240	0.083 ± 0.016	weak
105.1	1	0.058 ± 0.005	0.073	0.146	0.292	0.091 ± 0.019	weak	0.058 ± 0.005	0.073	0.146	0.291	0.327 ± 0.052	strong
106.3	4	0.058 ± 0.005	0.073	0.146	0.292	0.288 ± 0.069	moderate	0.006 ± 0.008	0.088	0.175	0.351	0.323 ± 0.109	moderate
106.5	1	0.063 ± 0.012	0.100	0.199	0.399	0.472 ± 0.068	strong	0.065 ± 0.002	0.070	0.141	0.281	0.430 ± 0.070	strong
107.1	4	0.063 ± 0.009	0.089	0.179	0.358	0.122 ± 0.017	weak	0.058 ± 0.004	0.070	0.141	0.282	0.148 ± 0.008	moderate
114.2	1	0.058 ± 0.007	0.080	0.159	0.319	0.350 ± 0.091	strong	0.062 ± 0.008	0.084	0.169	0.338	0.584 ± 0.128	strong
14018	1	0.060 ± 0.006	0.077	0.153	0.306	0.689 ± 0.084	strong	0.041 ± 0.003	0.050	0.099	0.198	1.088 ± 0.222	strong

[a]Four technical replicates of the negative control (containing no cells) in each independent experiment were included for each isolate and expressed as the mean OD_{492} ± standard deviation.

[b]The cut-off OD (ODc) was defined as three standard deviations (SDs) above the mean OD of the negative control.

[c]Eight technical replicates and four biological replicates (n=32) were performed for each isolate and the OD_{492} readings were averaged ±

standard deviation.

[d]Isolates were classified for their biofilm forming ability as described in Materials and Methods: no biofilm formers when $OD \leq ODc$, weak biofilm formers when $ODc < OD \leq 2 \times ODc$, moderate biofilm formers when $2 \times ODc < OD \leq 4 \times ODc$, strong biofilm formers when $4 \times ODc < OD$.

ND, not detected
