# Supplementary material for: Phenotypic characterization of Gardnerella vaginalis subgroups suggests differences in their virulence potential
Source: PLoS One. 2018 Jul 12;13(7):e0200625. doi: 10.1371/journal.pone.0200625 (PMC6042761; doi:10.1371/journal.pone.0200625)
Supplement: S1 File — (PDF) [file pone.0200625.s003.pdf]

Random amplified polymorphic DNA (RAPD) analysis of *G. vaginalis* isolates

The RAPD analysis [1, 2] was performed to disclose genetic diversity of *G. vaginalis* strains of the same clade isolated from the same vaginal sample. Multiple strains of clade 1 and clade 2 were isolated from the following samples: 084S1 yielded isolates 84.1 and 84.5 (both clade 1) and isolates 84.3, 84.4 and 84.6 (all clade 2); 086S1 yielded isolates 86.3 and 86.5 (both clade 2). All isolates of clade 4 analyzed in this study were also subjected for the RAPD analysis.

Two primers were used in RAPD reactions: G3-1F (5'-CAGATTAGCAGCAGCCGCC) and G5-1F (5'-GCGAGTGGGCTTGGTG). Amplification were performed in 25 µL reaction mixtures containing 1X Platinum Green Hot start PCR Master Mix (Thermo Fisher Scientific), 0.4 µM DNA primer, and 40 ng of genomic DNA. Amplification reactions included initial denaturation for 2 min at 94°C, 35 amplification cycles consisting of denaturation for 30 s at 94°C, annealing for 30 s at 38°C, and extension for 45 s at 72°C. The final extension step was prolonged for 7 min. PCR products (8 µL aliquots) were subjected to electrophoresis on a 1.2% agarose gel with subsequent staining with ethidium bromide and visualization under UV light.

RAPD fingerprints of the isolates of three clades are shown in the figure below. RAPD generated using two different primers produced strain-specific profiles among isolates, including those from samples 084S1 and 086S1 (A, B, C, D).

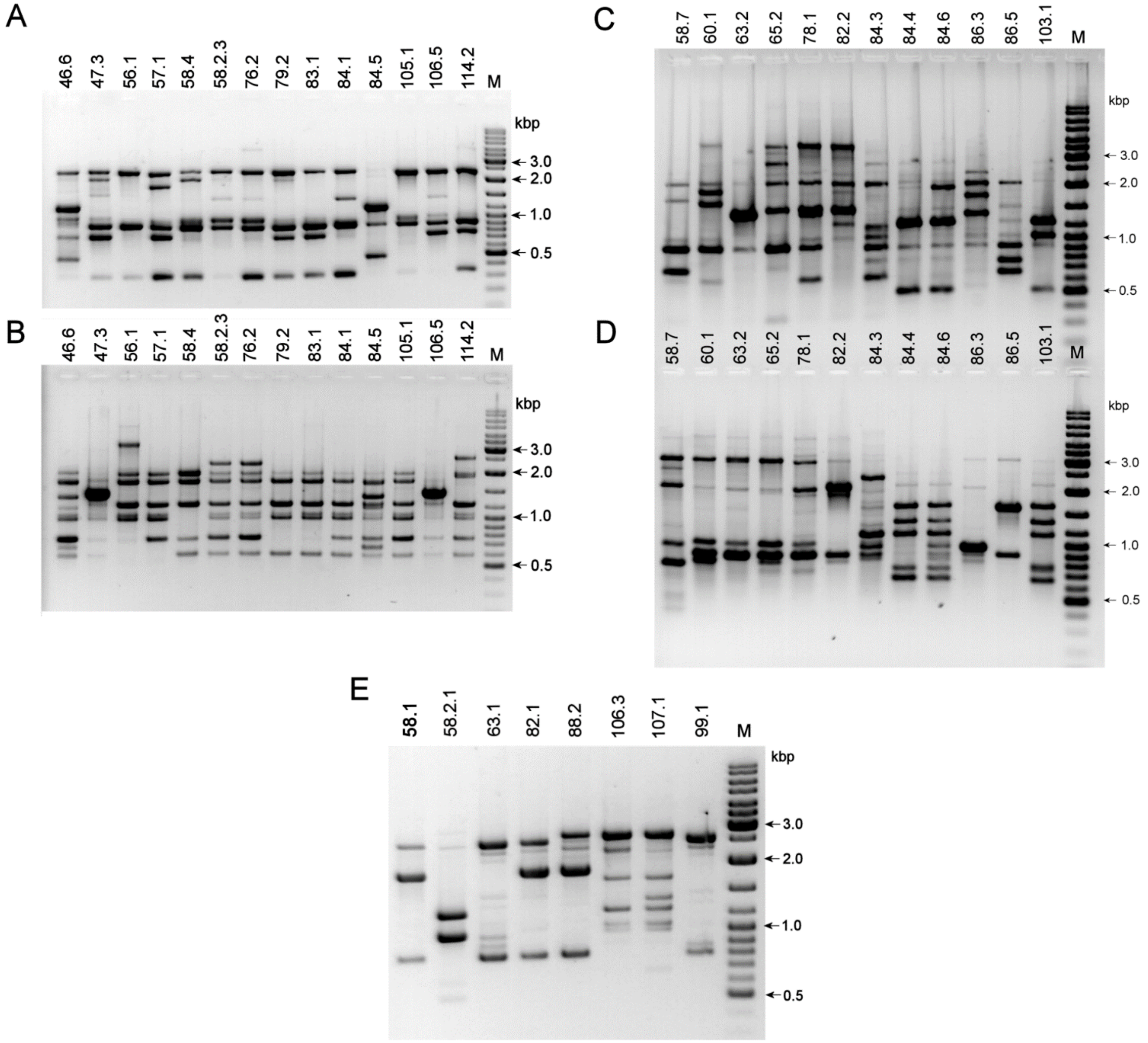

RAPD profiles of *G. vaginalis* isolates of clade 1 (A, B), clade 2 (C, D), and clade 4 (E) generated using primers 3G-1F (A, C) and G5-1F (B, D, E). The numbers of the isolates are indicated on the top. M, Gene Ruler DNA Ladder Mix (Thermo Fisher Scientific).

References

1. Macgowan AP, O'Donaghue K, Nicholls S, McLauchlin J, Bennet PM, Reeves DS. Typing of *Listeria* spp. by random amplified polymorphic DNA (RAPD) analysis. J Med Microbiol. 1993; 38: 322–327.
2. Akopyanz N, Bukanov NO, Westblom TU, Kresovich S, Berg DA. DNA diversity among clinical isolates of Helycobacter pylori detected by PCR-based RAPD fingerprinting. Nucleic Acids Res. 1992; 20: 5137–5142.
