# Supplementary material for: Phenotypic characterization of Gardnerella vaginalis subgroups suggests differences in their virulence potential
Source: PLoS One. 2018 Jul 12;13(7):e0200625. doi: 10.1371/journal.pone.0200625 (PMC6042761; doi:10.1371/journal.pone.0200625)
Supplement: S2 File — (A) Optical density (OD600) of G. vaginalis isolates after 24 h incubation. The growth assay of each isolate in liquid medium was performed as three independent experiments in duplicate. Error bars indicate standard deviation (SD). (B) The growth curves of isolates 58.7, 63.1, 84.3, 86.5, 88.2, and 99.1. Isolate 58.2.1was used as a control. The growth curves were generated from two independent experiments in duplicate as described previously [19]. Error bars indicate standard deviation (SD). (PDF) [file pone.0200625.s004.pdf]

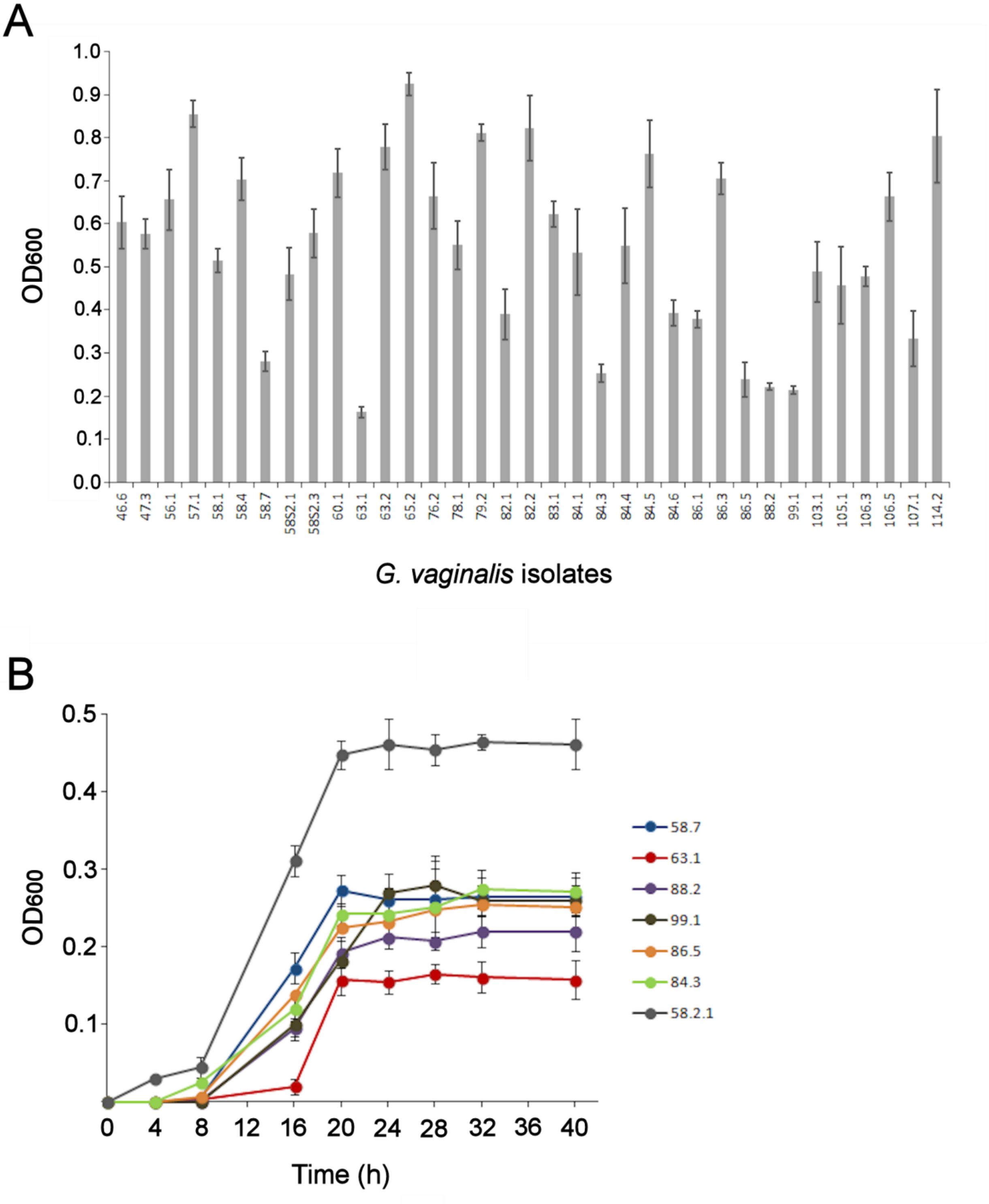

(A) Optical density (OD_{600}) of *G. vaginalis* isolates after 24 h incubation. The growth assay of each isolate in liquid medium was performed as three independent experiments in duplicate. Error bars indicate standard deviation (SD). **(B) The growth curves of isolates 58.7, 63.1, 84.3, 86.5, 88.2, and 99.1.** Isolate 58.2.1 was used as a control. The growth curves were generated from two independent experiments in duplicate as described previously [19]. Error bars indicate standard deviation (SD).
