# Supplementary material for: Phenotypic characterization of Gardnerella vaginalis subgroups suggests differences in their virulence potential
Source: PLoS One. 2018 Jul 12;13(7):e0200625. doi: 10.1371/journal.pone.0200625 (PMC6042761; doi:10.1371/journal.pone.0200625)
Supplement: S3 File — (PDF) [file pone.0200625.s005.pdf]

```
58.4Sia   392 ALFDTGYASSRSYRIPSLVKTRMGTLIAGADQRTSVSNDAPN-HINFVIRRSTDGGKTWKPLQTVINMPGKNLGKLGASAIDSCPILDPAGKSERINVLI
114.2Sia  392 ALFDTGYASSRSYRIPSLVKTRMGTLIAGADQRTSVSNDAPN-HINFVIRRSTDGGKTWKPLQTVINMPGKNLGKLGASAIDSCPILDPAGKSERINVLI
47.3Sia   392 ALFDTGYAGSRSYRIPSLVKTRMGTLIAGADQRTSVSNDAPN-HINFVIRRSTDGGKTWKPLQTVINMPGKNLGKLGASAIDSCPILDPAGKSERINVLI
79.2Sia   392 ALFDTGYAGSRSYRIPSLVKTRMGTLIAGADQRTSVSNDAPN-HINFVIRRSTDGGKTWKPLQTVINMPGKNLGKLGASAIDSCPILDPAGKSERINVLI
60.1Sia   395 ALFDTGYAGSRSYRIPSLVKTHMGTLIAGADQRTSVSNDAPN-HINFVIRRSADGGKTWGPLQTVINMPGKNLGKLGASAIDSCPVLDPEGKSKRINVLI
86.1Sia   386 ALFDTGYAQSRSYRIPSLVKTHMGTLIAGADQRTSVSNDAPN-HINFVIRRSTDGGKTWGPLQTVINMPGKNLGKLGASAIDSCPILDPAGKSERINLLI
Cper.Sia  253 DLFHPGFLNSSNYRIPALFKTKEGTLIASIDARRHGGADAPNNDIDTAVRRSEDGGKTWDEGQIIMDYPDK------SSVIDTTLIQD--DETGRIFLLV
                           ■                        ●            ═════════

58.4Sia   491 DLNPSGIGLTNCKTAVGVDACGRIKLIDRLGGNYAAAMDGSAAVKLPCDSSEKECATKWLVFPDGSIKNCDSHNDLRN----NSHSKQTCDSSTWNIWKS
114.2Sia  491 DLNPSGIGLTNCKTAVGVDACGRIKLIDRLGGNYAAAMDGSAAVKLPCDSSEKECATKWLFFPDGSIKNCDSHNDLRN----NSHSKQTCDSSTWNIWKS
47.3Sia   491 DLNPSGIGLTNCKTAVGIDACGRIKLIDRLGGNYAAAMDGSAAVKLPCDSSEKECATKWLVFPDGSIKNCDSHNDLRN----NSHSNQTCDSSTWNIWKS
79.2Sia   491 DLNPSGIGLTNCKTAVGIDACGRIKLIDRLGGNYAAAMDGSAAVKLPCDSSEKECATKWLVFPDGSIKNCDSHNDLRN----NSHSNQTCDSSTWNIWKS
60.1Sia   494 DINPGGIGLTNCKTAIGVDACGRIKLIDRLGGNYAAAMDASAAVKLPCDSSEKECATKWLVFPDGSIKNCDSHDDLNDDSKINSHSNQTCDCSTWNIWKS
86.1Sia   485 DLNPSGIGLTNCKTAVGVDECGRIKLEDRFGIHYSAAMNGSAAVKLPCDSSEKECATKWLVSPDGSIKNCDSHNDSRN----NSHSNQTCDSSTWNIWKS
Cper.Sia  345 THFPSKYGFWNAGLGSGFKNID--------GKEYLCLYD----------SSGKEFTVRENVVYDKDGNKTEYTTNALG------------DLFKNGTKI

58.4Sia   587 QEIASESQPLFAEKTCYIAQIYSDDDGKTWSVPRLIDHMVKEPWMSFAGVCPGNGIVIRQSEKHYGRILVPFYCSGKSKSHYSSGALISDDDGKTWSRGK
114.2Sia  587 QEIASESQPLFAEKTCYIAQIYSDDDGKTWSVPRLIDHMVKEPWMSFAGVCPGNGIVIRQSEKHYGRILAPFYCSGKSKSHYSSGALISDDDGKTWSRGK
47.3Sia   587 QEIASESQPLFAEKTCYIAQIYSDDDGKTWSVPRLIDHMVKEPWMSFAGVCPGNGIVIRQSEKHYGRILAPFYCSGQSKSHYSSGALISDDEGKTWSRGK
79.2Sia   587 QEIASESQPLFAEKTCYIAQIYSDDDGKTWSVPRLIDHMVKEPWMSFAGVCPGNGIVIRQSEKHYGRILAPFYCSSQSKSHYSSGALISDDEGKTWSRGK
60.1Sia   594 PEIASESQPLFAEKTCYIAQIYSDDDGKTWSAPRLIDHMVKEPWMSFAGVCPGNGIVIRHSEKHYGRILVPFYCSGQSKSHYSSGALISDDEGKTWSRGK
86.1Sia   581 QEIASESQPLFAEKTCYIAQIYSDDDGKTWSAPRLIDHMVKEPWMSFAGLCPGNGIVIRQSEKHYGRILVPFYCSGKSKSHYSSGALISDDDGKTWSRGK
Cper.Sia  414 DNINSSTAPLKAKGTSYINLVYSDDDGKTWSEPQNINFQVKKDWMKFLGIAPGRGIQIKNGE-HKGRIVVPVYYTNE-KGKQSSAVIYSDDSGKNWTIGE
                                    ═════════                                                         ═════════

58.4Sia   687 MINEGR-LINGKIVDPVTMQDDDATSSETVFVERKNGDILAFFRNQNRSGCVGKAISHDCGETWSELIFDTS-LPEIFSQPSATCFNSEN---AENADCI
114.2Sia  687 MINEGR-LINGKIVDPVTMQDDDATSSETVFVERKNGDILAFFRNQNRSGCVGKAISHDCGETWSELIFDTS-LPEIFSQPSATCFNSEN---AENADCI
47.3Sia   687 MINEGR-LINGKIVDPVTMQDDDATSSETVFVERKNGDILAFFRNQNRSGCVGKAISHDCGETWSELIFDTS-LPEIFSQPSATCFNSEN---ADNADCI
79.2Sia   687 MINEGR-LINGKIVDPVTMQDDDATSSETVFVERKNGDILAFFRNQNRSGCVGKAISHDCGETWSELIFDTS-LPEIFSQPSATCFNSEN---ADNADCI
60.1Sia   694 MINEGR-LINGKIVDPATMQDDDATSSETVFVERKNGDILAFFRNQNRSGCVGKAISHDCGETWSELIFDTS-LPEIFSQPSATCFSSENDRNAQNADCI
86.1Sia   681 MINEGR-SINGRIVDPVTMQDDDATSSETVFVERKNGDILAFFRNQNRSGCVGKAISHDCGETWSELIFDTS-LPEIFSQPSATCFNSEN---ADNADCI
Cper.Sia  512 SPNDNRKLENGKIINSKTLSDDAPQLTECQVVEMPNGQLKLFMRN--LSGYLNIATSFDGGATWDETVEKDTNVLEPYCQLSVINYSQKV----DGKDAV
                                         ○               ■            ═════════

58.4Sia   782 AFANASQMMPYRGRGVVRFS-----YDGGK-----TWAKNVCINPFHHVYQCLSSSNKRTLQLLWERETTGIYITAIDSNL
114.2Sia  782 AFANASQMMPYRGRGVVRFS-----YDGGK-----TWAKNVCINPFHHVYQCLSSSNKRTLQLLWERETTGIYITAIDSNL
47.3Sia   782 AFANASQMMPYRGRGVVRFS-----YDGGK-----TWAKNVCINPFHHVYQCLSSSNKRTLQLLWERETTGIYITAIDSNL
79.2Sia   782 AFANASQMMPYRGRGVVRFS-----YDGGK-----TWAKNVCINPFHHVYQCLSSSNKRTLQLLWERETTGIYITAIDSNL
60.1Sia   792 AFANASQMMPYRGRGVVRFS-----YDGGK-----TWAKSVCINPFHHVYQCLSSSNKRTLQLLWERETTGIYITAIDSSL
86.1Sia   776 AFANASQMMPYRGRGVVRFS-----FDGGK-----TWAKNVCINPFHHVYQCLSSSNKRTLQLLWERETTGIYITAIDSNL
Cper.Sia  606 IFSNPNARSRSNGTVRIGLINQVGTYENGEPKYEFDWKYNKLVKPGYYAYSCLTELSNGNIGLLYEGTPS-EEMSYIEMNL
                         ■       =      ═════    ═══           ▲               □
```

Alignment of amino acid sequences of sialidase catalytic domain from *G. vaginalis* isolates 47.3, 58.4, 60.1, 79.2, 86.1 and 114.2. Cper indicates NanI exo-α-sialidase of *Clostridium perfringens* (GenBank CAA60796.1; [1]). *C. perfringens* sialidase had the best match with *G. vaginalis* sialidases according to the Protein Data Bank for the solved structures [2]. The sequences were aligned using ClustalW [3]. The

conserved motives of *G. vaginalis* sialidases were marked according to NCBI Conserved Domain Database (CDD) [4] and previous studies [5] as follows: identical residues, black background; tri-arginine cluster, closed square (■); acid/base aspartic acid, closed circle (●); BNR/Asp-box motifs, double line (═) ; nucleophilic tyrosine, closed triangle (▲); conserved in CDD, open circle (○); glutamic acid stabilizing the tri-arginine cluster, open square (□).

References

1. Newstead SL, Potter JA, Wilson JC, Xu G, Chien CH, Watts AG, et al. The structure of *Clostridium perfringens* NanI sialidase and its catalytic intermediates. J Biol Chem. 2008; 283: 9080–9088. doi:10.1074/jbc.M710247200.
2. Pieper U, Webb BM, Dong GQ, Schneidman-Duhovny D, Fan H, Kim SJ, et al. MODBASE, a database of annotated comparative protein structure models and associated resources. Nucleic Acids Res. 2013; 42: D336–D346. doi: 10.1093/nar/gkt1144.
3. Aiyar A. The use of CLUTAL W and CLUSTAL X for multiple sequence alignment. Methods Mol Biol. 2000; 132: 221–241. Available from: https://npsa-prabi.ibcp.fr/cgi-bin/npsa_automat.pl?page=npsa_clustalw.html.
4. Marchler-Bauer A, Bo Y, Han L, He J, Lanczycki CJ, Lu S, et al. CDD / SPARCLE : functional classification of proteins via subfamily domain architectures. Nucleic Acids Res. 2017; 45: D200–D203. doi:10.1093/nar/gkw1129.
5. Kiyohara M, Tanigawa K, Chaiwangsri T, Katayama T, Ashida H, Yamamoto K. An exo-alpha-sialidase from bifidobacteria involved in the degradation of sialyloligosaccharides in human milk and intestinal glycoconjugates. Glycobiology 2011; 21: 437–447. doi:10.1093/glycob/cwq175.
