# Supplementary material for: Phenotypic characterization of Gardnerella vaginalis subgroups suggests differences in their virulence potential
Source: PLoS One. 2018 Jul 12;13(7):e0200625. doi: 10.1371/journal.pone.0200625 (PMC6042761; doi:10.1371/journal.pone.0200625)
Supplement: S1 Fig — G. vaginalis isolates 84.5, 86.3, and 88.2 were vly positive, whereas isolates 58.7, 84.3, 84.4, 84.6, 86.1, and 86.5 were vly negative. M, Gene Ruler DNA Ladder Mix (Thermo Fisher Scientific). The PCR results were verified by the ELISA quantification of VLY in bacterial supernatants. (PDF) [file pone.0200625.s006.pdf]

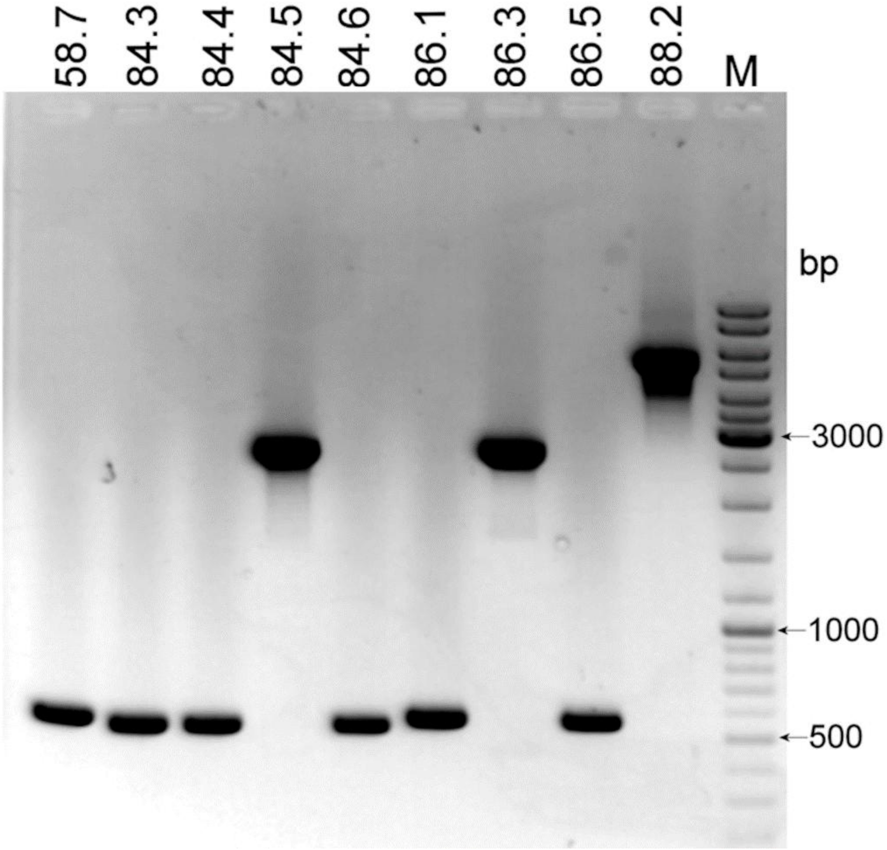

S1 Fig. Amplification of *vly* gene using primers flanking its region. *G. vaginalis* isolates 84.5, 86.3, and 88.2 were *vly* positive, whereas isolates 58.7, 84.3, 84.4, 84.6, 86.1, and 86.5 were *vly* negative. M, Gene Ruler DNA Ladder Mix (Thermo Fisher Scientific). The PCR results were verified by the ELISA quatification of VLY in bacterial supernatants.
